# Elevated B12: A Cross-Sectional Survey of Disease Associations and Attitudes of General Practitioners and Medical Students

**DOI:** 10.7759/cureus.99690

**Published:** 2025-12-20

**Authors:** Hamza A Mahmood, Pratima Singh, Himanshu Tyagi

**Affiliations:** 1 Medicine, Frimley Health NHS Foundation Trust, Slough, GBR; 2 Psychiatry, Hertfordshire Partnership NHS Foundation Trust, Hatfield, GBR; 3 Neuropsychiatry, National Hospital for Neurology and Neurosurgery, London, GBR

**Keywords:** b12 pseudo-hypervitaminosis, elevated b12, general practitioners, hypercobalaminaemia, primary care awareness, pseudo-hypercobalaminaemia

## Abstract

Introduction

Elevated serum B12 (hypercobalaminaemia) has been linked to malignancies, liver disease, and increased mortality. Two additional concepts complicate interpretation. B12 pseudo-hypervitaminosis is a paradoxical state of elevated B12 in serum but intracellularly unavailable, leading to functional deficiency and B12-deficiency symptoms. Pseudo-hypercobalaminaemia is a laboratory artefact, with elevated B12 detected despite true low levels. Despite significant clinical implications, raised B12 is overlooked in primary care and medical education.

This pilot study aims to assess the awareness of general practitioners (GPs) and medical students regarding the clinical implications of elevated serum B12.

Methods

A cross-sectional survey of 46 UK-based GPs and 50 medical students was conducted electronically using convenience sampling. The survey assessed candidates’ awareness of causes, consequences, investigations, and management of high serum B12.

Results

Among 46 GPs, 41 (89.2%) recognised at least one disease association with hypercobalaminaemia compared to 11 (22%) of 50 medical students (p < 0.0001). Common associations included malignancy, haematological conditions, and liver pathology. Neither group demonstrated awareness of pseudo-hypercobalaminaemia or B12 pseudo-hypervitaminosis. A total of 30 (65%) GPs and 27 (54%) medical students would investigate elevated B12, yet these approaches were highly variable and frequently inappropriate. Awareness of disease associations significantly increased the likelihood of choosing an appropriate investigation (p < 0.0001). Most respondents reported receiving no formal teaching on elevated B12 levels. GPs and medical students were similar in their support for changing cancer guidelines to reflect hypercobalaminaemia, yet awareness of disease links did not significantly impact support. The qualification date of GPs did not significantly impact their awareness, investigative approaches, or attitude towards guideline changes.

Conclusions

This pilot survey demonstrates a clear gap in clinician understanding of hypercobalaminaemia and the lack of standardised investigative approaches. Larger, multi-centred studies are needed to develop and validate practical diagnostic pathways for primary care.

## Introduction

Vitamin B12 testing is commonly requested in primary care to assess for deficiency. However, elevated B12 (hypercobalaminaemia) is often overlooked or poorly understood, despite its clinical implications [1]. There is no recognised toxicity state (i.e., no true hypervitaminosis), and the most common cause of elevated B12 is supplementation. There are, however, multiple disease associations. There is evidence of association between hypercobalaminaemia and mortality [2-5], liver disease (including acute hepatitis, liver cirrhosis, cystic fibrosis-mediated liver disease, and hepatocellular carcinoma) [6-9], malignancies (such as prostate and breast cancer, liver tumours, lymphomas, and metastatic disease) [10-12], and haematological conditions, including chronic myeloid leukaemia, polycythaemia vera, hypereosinophilic syndrome, myelofibrosis, and acute promyelocytic leukaemia [13-16].

Interpretation is further complicated by two related concepts. Pseudo-hypervitaminosis is a phenomenon that describes a functional deficiency despite true B12 elevation, due to decreased intracellular availability of B12. This may lead to symptoms of B12 deficiency, which clinicians may overlook as the laboratory values appear paradoxically elevated [17]. Pseudo-hypercobalaminaemia is an additional phenomenon that refers to falsely elevated serum B12 levels due to an assay artefact, which can occur due to the presence of immune complexes [18,19]. This can have similar effects to pseudo-hypervitaminosis with falsely elevated laboratory values [20,21].

Diagnosis of hypercobalaminaemia is difficult as there is a lack of universally agreed reference ranges, with most laboratories using thresholds of around >900-950 pg/mL (660-700pmol/L). Similarly, this is difficult because different laboratory assays would give different values of B12 for the same sample [22,23]. Standard assays measure total circulating B12, including fractions bound to haptocorrin, transcobalamin, and immunoglobulins, rather than the biologically active component [24]. A multimarker approach incorporating functional indices such as methylmalonic acid (MMA), homocysteine, and carrier proteins (transcobalamin, haptocorrin) would be an alternative approach, which would better identify functional deficiency and distinguish laboratory artefacts [25-28].

Unlike B12 deficiency, which has clear guidelines, there is no standardised approach for investigating or managing hypercobalaminaemia, creating clinical uncertainty. The epidemiology of hypercobalaminaemia is poorly characterised, with no current large-scale epidemiological datasets showing patients with hypercobalaminaemia who have concurrent neurological symptoms of deficiency. However, existing evidence does suggest that normal or high serum B12 does not reliably exclude deficiency [26,29].

As most serum B12 tests are organised by general practitioners (GPs), their understanding and response to elevated results is crucial. Elevated B12 is not uncommon at the population level. Data from the National Health and Nutrition Examination Survey (NHANES) from 1999 to 2014 showed 6.9% of adults had serum B12 above 700 pmol/L [23]. The primary objective of this study, therefore, was to assess GPs’ awareness of elevated B12 in terms of its disease associations, their investigations, management, and understanding of pseudo-hypervitaminosis or pseudo-hypercobalaminaemia. By improving primary care education, patient outcomes can improve by possibly identifying neuropsychiatric conditions and malignancies earlier. The secondary objective of this study is to assess if current medical education addresses this subject, so we have also included an additional survey for medical students.

This article was previously presented as an oral abstract presentation at the British Neuropsychiatry Association 38th Annual Conference on 13 March 2025 and a poster presentation at the Faculty of Liaison Psychiatry Annual Conference on 14 May 2025 and the Royal College of Psychiatrists International Congress on 23-26 June 2025.

## Materials and methods

Study design

Two cross-sectional surveys were conducted to explore awareness and clinical approaches to elevated B12. These were sent out to practising GPs and medical students in the UK. Both surveys were administered using Google Forms (Google, Mountain View, CA).

The survey was carried out from April to May 2024 for GPs (Table 1) and from October to November 2024 for medical students (Table 2). The questionnaires involved eight items for GPs and five items for medical students. Basic demographic information from GPs was collected, including year of graduation, year of qualifying as a GP, and medical school attended.

**Table 1:** Survey questions for general practitioners (GPs).

Question
What country did you study medicine in?
What year did you graduate from medicine?
What year did you qualify as a GP?
Which university did you attend?
Would you investigate a patient with high B12 levels? If yes, how so?
What conditions can you think of that are associated with high B12 levels?
Do you expect cancer referral guidelines to change to incorporate patients with high B12 levels?
Did your medical school give any teaching on high B12?

**Table 2:** Survey questions for medical students.

Question
What year of medicine are you in?
Has your medical school given you any teaching on high B12? If yes, what teaching have they provided?
Would you investigate a patient with high B12 levels? If yes, how would you investigate them?
Can you list any conditions associated with high B12 levels?
Would you expect cancer referral guidelines to change to incorporate patients with high B12 levels?

Ethical considerations

As the surveys involved anonymised responses from clinicians and medical students, and did not collect any patient data, formal ethical approval was not deemed required in accordance with the UK Health Research Authority guidance for service evaluation and educational surveys. A standardised message was sent alongside the survey link to inform participants of the study’s purpose, and consent was implied by voluntary completion of the questionnaires. Although names and email addresses were initially collected to verify eligibility and prevent duplicate responses, these identifiers were not analysed, and all data were anonymised prior to analysis. Participation was therefore voluntary, and results are anonymous.

Recruitment

Recruitment for the surveys was performed using a multimodal approach. GPs were recruited via convenience sampling through professional networks. Five GP partners and a salaried GP in the South East of England were contacted and asked to distribute the survey link to colleagues within their workplace and networks. The survey was included as part of a standardised message that was sent, explaining the rationale behind the survey. Based on their estimates and practice sizes, the survey reached around 80-120 clinicians, and 46 responses were achieved (estimated response of 38-58%).

Medical students were also recruited via convenience sampling. A standardised message including the survey link was sent to relevant social media group chats (418 members), and individual messages were also sent to colleagues, reaching approximately 450 members. A total of 50 responses were returned with an estimated response rate of 11%. The majority of students were from clinical years of medicine, and eight were from pre-clinical years.

Survey development

The survey questions were developed to evaluate awareness of elevated B12, perceived clinical significance, investigation and management strategies, and potential disease associations. We also wanted to assess if there was awareness of functional deficiency due to pseudo-hypervitaminosis. The questions were developed alongside a consultant neuropsychiatrist for clinical relevance and clarity. The questionnaires underwent pilot testing with a small cohort (n = 10) of medical students and GPs to assess question suitability and readability prior to distribution. Based on feedback, adaptations were made to the survey for readability purposes.

Definitions and coding

The surveys contained a mixture of open-ended and reply-option (yes/no) items. In the GP survey, demographic questions (country of study, year of graduation, year of GP qualification, and medical school attended) were short-text factual items, which did not require coding. The teaching question was a yes/no response. The item asking whether cancer guidelines should change was open-ended, but all responses were given in a yes/no (or synonymous) format; therefore, no additional coding was needed.

Open-ended questions requiring coding were used for listing disease associations of elevated B12 and describing investigative approaches.

Awareness was defined as a correct identification of at least one clinical association that is recognised in the literature. This included malignancy, haematological malignancy/myeloproliferative neoplasm, liver disease, or renal failure. For investigative approaches, we coded “would investigate elevated B12” only if a response included any action beyond repeating the test or asking about supplements. These included ordering blood tests, screening for malignancy, a referral (i.e., to haematology or secondary care), imaging, and investigating after confirming no supplementation. Responses that involved only checking supplementation or repeating the test were coded as “would not investigate elevated B12”, as these actions, while sensible for excluding erroneous results, do not themselves constitute evaluation of secondary, pathological causes.

For medical students, the format was adapted to include initial reply-option questions with optional open-text follow-up. Teaching and guideline-change questions were yes/no items. Both awareness and investigative approaches followed the same coding as above.

Statistical analysis

The sample size was chosen for feasibility purposes, given the small research group. Given that there is a lack of any existing data on clinician awareness of raised B12 levels, particularly among primary care physicians, this study was designed as an exploratory, hypothesis-generating investigation using a pragmatic convenience sample. The findings are not intended to be generalisable, but are to inform whether larger, multi-centre research would be warranted. As this is an exploratory study, a survey including 96 participants was viewed as sufficient.

Categorical variables were summarised as frequencies and percentages. Differences between GPs and medical students were analysed using chi-square or Fisher’s exact tests when expected cell counts were below five. We compared GPs and medical students in terms of awareness of clinical associations, investigative approaches, previous teaching experience, and support for guideline changes. Effect sizes were expressed as Cramer’s V and odds ratios (ORs), with 95% confidence intervals. For the primary comparison of awareness of clinical associations of elevated B12 among GPs and medical students, we calculated Cohen’s h for the difference in proportions, with our value exceeding the conventional threshold for a large effect of 0.8. Given the observed effect size and total sample of 96 participants, this primary analysis had very high statistical power of >0.99 at alpha = 0.05. No formal a priori power calculation was required, given that this was an exploratory feasibility study.

The questionnaires were not designed as psychometric scales; hence, Cronbach’s alpha was not calculated. The aim was to assess awareness and feasibility rather than to develop a validated measure.

## Results

A total of 96 responses were analysed, comprising 46 GPs and 50 medical students, with results summarised in Table 3.

**Table 3:** Comparison of the awareness and practices of general practitioners (GPs) (n = 46) and medical students (n = 50) regarding elevated B12 levels.

Category	GPs (n = 46)	Medical students (n = 50)
Identified a disease associated with elevated B12	41 (89.2%)	11 (22%)
Would investigate high B12	30 (65%)	27 (54%)
Would support an update for referral guidelines	17 (36.9%)	18 (36%)
Received no education on the consequences of high B12	34 (73.9%)	41 (82%)

Awareness

**Figure 1:**
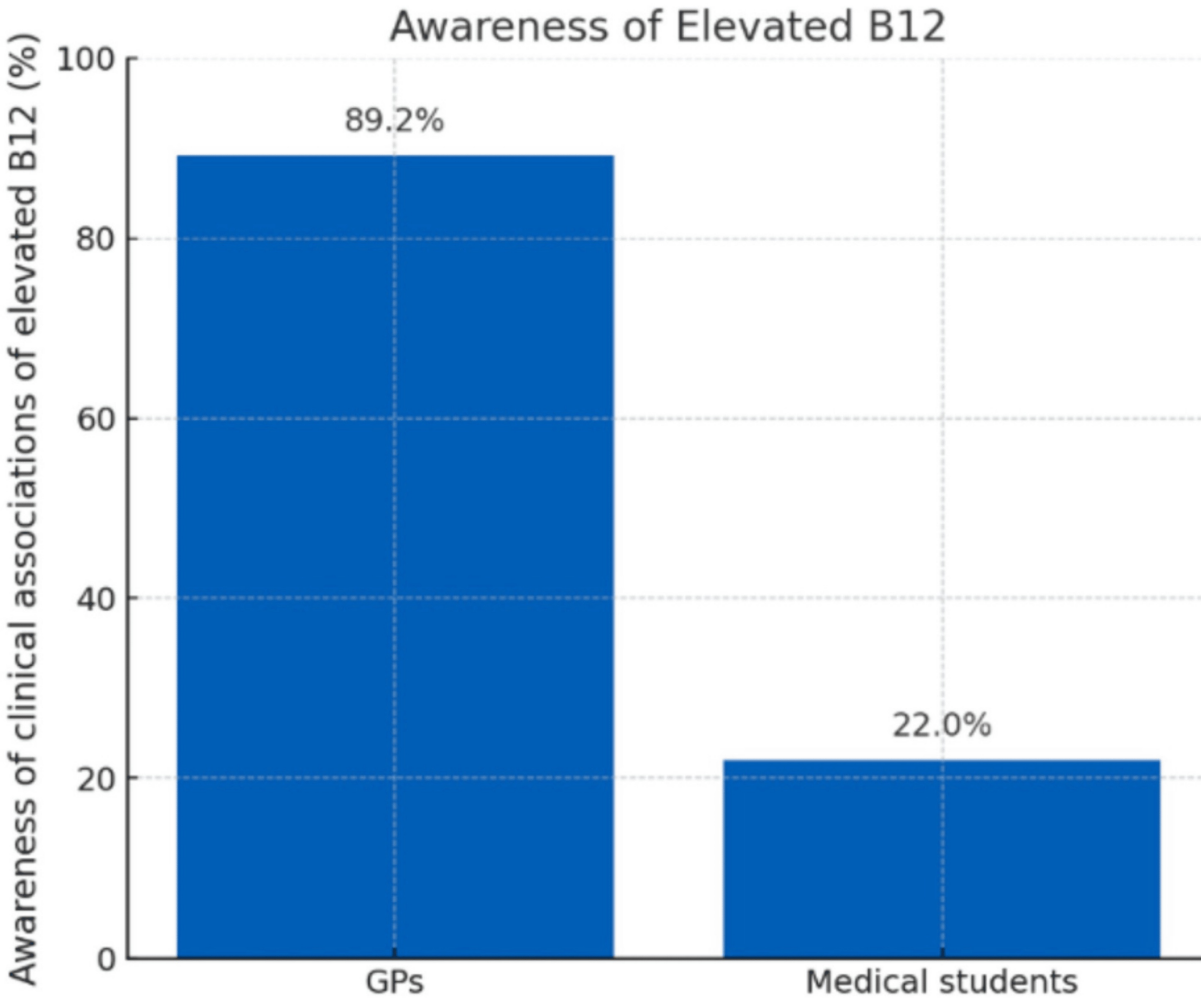
Awareness of clinical associations of elevated B12 among GPs and medical students. A significantly higher proportion of GPs recognised at least one clinical association of elevated B12 compared to medical students (89.2% vs. 22.0%; χ²(1) = 43.49, p < 0.0001; Cramer’s V = 0.67, Cohen’s h = 1.5). GPs: general practitioners.

**Figure 2:**
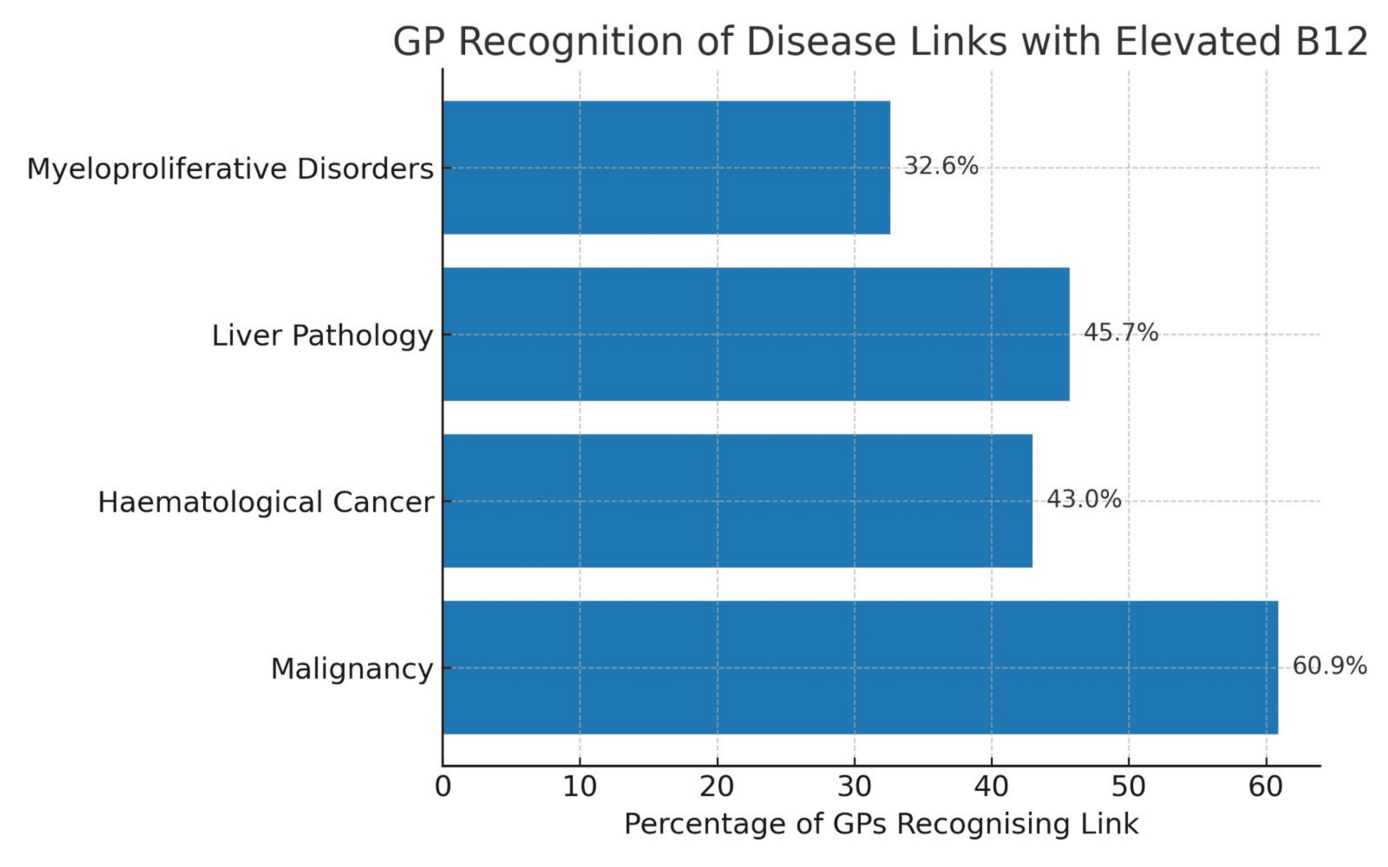
GPs' recognition of disease links with elevated B12. GPs: general practitioners.

Awareness differed markedly between the cohorts, with results shown in Figure 1. A total of 41 (89.2%) of 46 GPs recognised at least one disease association compared to only 11 (22%) of 50 medical students. This difference was statistically significant with a large effect size (χ²(1) = 43.49, p < 0.0001; Cramer’s V = 0.67, Cohen’s h = 1.5). Among GPs, malignancy was the most frequently identified disease by 28 (60.9%) and haematological cancers were identified by 20 (43%). Elevated B12 was also linked to liver pathology by 21 (45.7%) and myeloproliferative disorders by 15 (32.6%), as illustrated in Figure 2. No respondent in either cohort mentioned mortality risk, pseudo-hypervitaminosis, or pseudo-hypercobalaminaemia.

Investigations

Among GPs, 30 (65%) would investigate, and among students, 27 (54%) would investigate elevated B12 levels. The difference here was not statistically significant (χ²(1) = 1.22, p = 0.27; Cramer’s V = 0.11). Although 27/50 medical students stated they would investigate, their choice of investigations was often limited or inappropriate, with vague answers of “blood tests” or “refer to a specialist”, or suggestions like “spine MRI”, with no clear justification. Clear and appropriate investigations were provided by eight of the 27 medical students and 21 out of the 30 GPs. Among GPs, the most common investigation of choice was blood tests (16/30, 53%), most commonly a full blood count (FBC). Of the GPs who would investigate, six (20%) would be looking for evidence of cancer.

**Figure 3:**
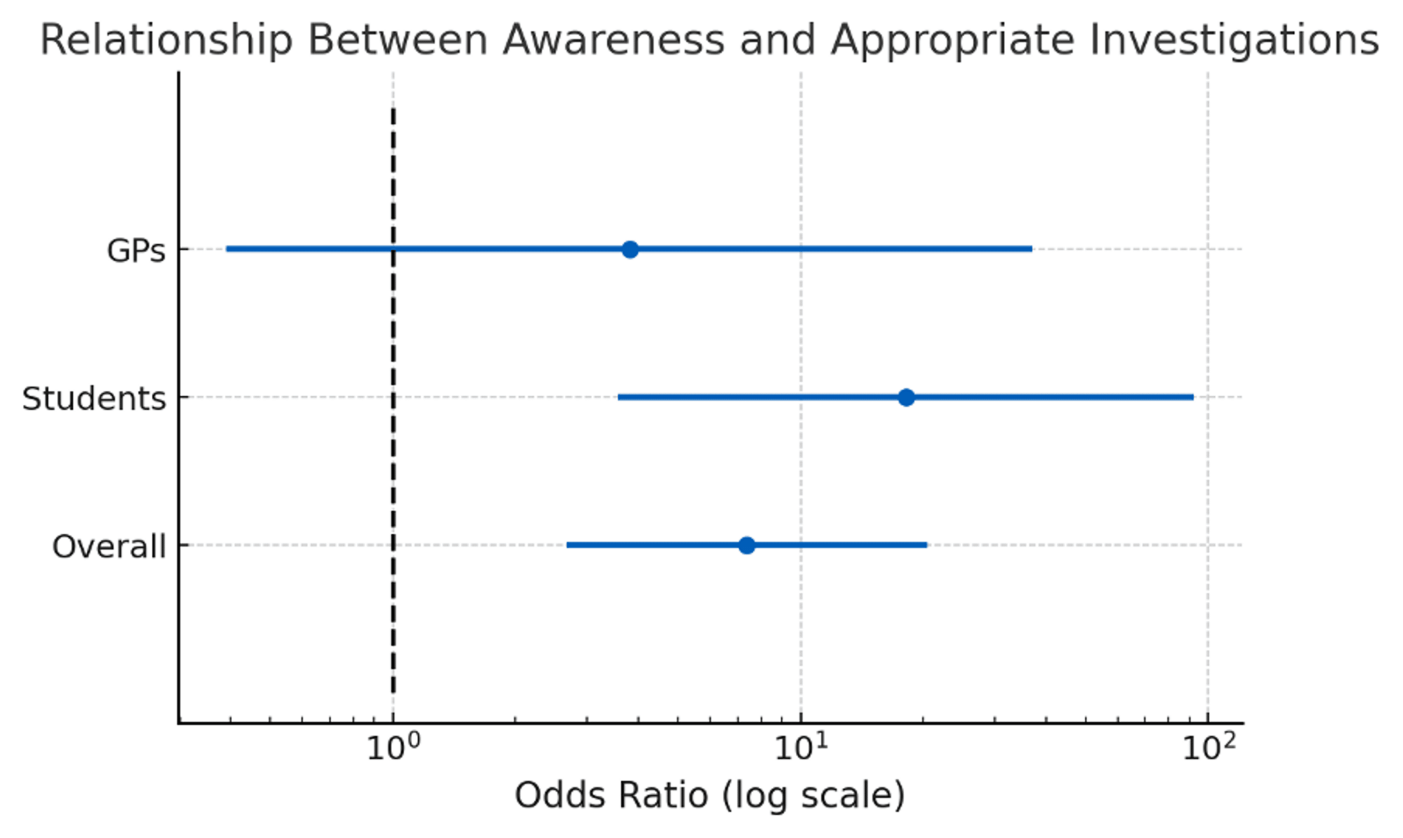
The impact of awareness on appropriate investigations for GPs, students, and combined. An appropriate investigation was defined as a clear, unambiguous next step that was not just simply repeating results or clarifying supplementation use. The association was significant for the combined group and for students alone, but not for GPs alone (Fisher’s exact test: combined, p < 0.0001; students, p < 0.0002; GPs, p = 0.36). GPs: general practitioners.

Among all respondents, awareness of an associated condition was significantly related to investigation behaviour, highlighted in Figure 3. Those who demonstrated awareness among GPs and medical students were significantly more likely to appropriately investigate than those without awareness (Fisher’s exact test, OR = 7.39, p < 0.0001). Further subgroup analysis showed that this difference was significant for medical students (Fisher’s exact test, OR = 18.13, p < 0.0002) but not for GPs (Fisher’s exact test, OR = 3.81, p = 0.36). The lack of significance among GPs is likely due to the low sample size of GPs who were not aware of disease associations, rather than the absence of a true effect.

Teaching

Part of our survey had GPs and medical students recall if they had been taught about high levels of B12 as part of their medical school. Among GPs, 34 (73.9%) received no such teaching, and neither did 41 (82%) of medical students. There was no significant difference between the proportion of GPs or medical students who had teaching (Fisher’s exact test, OR = 1.61, p = 0.46). Medical students were also asked explicitly what type of teaching they had. All free-text responses were linked to B12 deficiency, and none mentioned diseases associated with high serum B12, pseudo-hypervitaminosis, or pseudo-hypercobalaminaemia. Hence, this suggests that perceived teaching on B12 was more likely a misinterpretation of teaching on deficiency, rather than a true education on elevated B12.

Cancer guidelines

Support from GPs and medical students was similar in favour of changing cancer guidelines to reflect hypercobalaminaemia (in favour: 17 GPs, 36.9%; 18 medical students, 36%; Fisher’s exact test, OR = 1.07, p = 1). Among both GPs and medical students, awareness did not significantly impact support for changing cancer guidelines (Fisher’s exact test, OR = 1.75, p = 0.32). With further subgroup analysis, neither GPs (Fisher’s exact test, OR = 5.7 using Haldane-Anscombe correction, p = 0.31) nor medical students (Fisher’s exact test, OR = 1.67, p = 0.53) showed a significant association of knowledge with support of guideline change. The lack of significance among GPs was perhaps due to the low sample size of GPs who were not aware of disease associations, rather than a true effect.

Impact of qualification date

The median year of graduation of GPs was 2008. There was no significant difference between GPs who graduated earlier and later in terms of awareness (Fisher’s exact test, OR = 0.63, p = 1), numbers who would investigate (Fisher’s exact test, OR = 0.303, p = 0.07), or those who would support cancer guideline changes to reflect hypercobalaminaemia (Fisher’s exact test, OR = 1.21, p = 1).

## Discussion

Summary of findings

In this exploratory survey, most GPs were aware of the link between hypercobalaminaemia and disease, including liver pathology [6-9] and malignancy [10-12]. From our data, there was little awareness of functional deficiency (pseudo-hypervitaminosis) [17] or the possibility of artefacts (pseudo-hypercobalaminaemia) [18-21]. GPs were also significantly more aware of disease associations than medical students, with both groups receiving a predominant lack of teaching in this area. This suggests that it is likely through practice experience that common associations are seen. Furthermore, while medical students had a low rate of disease awareness, more than half opted to investigate. Most investigation suggestions were not appropriate; however, they suggested uncertainty in interpretation, rather than clinical reasoning. Among GPs and medical students, those with greater awareness of disease are understandably more likely to investigate hypercobalaminaemia. Whether a GP graduated more recently did not have a significant impact on their attitudes or awareness of hypercobalaminaemia. This suggests that limited formal teaching on elevated B12 is likely a persistent issue across both historical and contemporary medical education, rather than a problem solely confined to earlier training eras. While a minority of medical students did report having had teaching on hypercobalaminaemia, it was apparent through the free-text option that there was confusion between B12 deficiency and hypercobalaminaemia.

Strengths and limitations

The study gathered responses from a heterogeneous group of GPs, trained in different international systems, with a small number of GP trainees included. While the diversity may reflect global variation in training and reference ranges for B12 [22,23], the small numbers from each country limited meaningful comparison. The sample size was modest and relied on convenience sampling, which would reduce generalisability; however, the post-hoc power calculation showed that the sample size was sufficient. Free-text responses provided qualitative insights, with attitudes of GPs and medical students suggesting that this was an area of confusion, and further guideline support would be warranted. Despite this, by using free-text, some responses were difficult to categorise and lacked clarity, thereby limiting interpretation. An example of this was when asked if they would investigate, one GP wrote “depends if supplementation”, without a clear indication of how they would investigate or under what conditions. Additionally, the study was subject to the typical biases of a survey, namely, selection and recall bias, asking GPs who may have qualified decades ago about their medical school education. To attempt to alleviate this, we also included the attitudes of current medical students to identify if there is an update to the curriculum. As is possible with survey-based questionnaires, our question order may have introduced response bias. This could have been the case when participants were asked for disease associations and whether cancer referral guidelines ought to be updated. This could have primed participants to list cancer as a disease association even if they were not previously aware of this. Despite this, however, only one medical student and three GPs listed solely cancer or malignancy as a disease association; hence, this effect, if present, was minimal. Through the free text approach of the survey, we were able to ascertain the opinions of the students, such as one stating: “We’ve only been taught deficiency though so have no idea what I’d do”. It cannot be ruled out, however, that attitudes may have developed since the survey was conducted, due to the lack of follow-up. As an exploratory pilot, this study aimed to identify whether a meaningful knowledge gap exists. Now that, from the survey, this has been demonstrated, larger multi-centre studies with structured questions and follow-up would strengthen future recommendations.

Comparison with existing literature

The existing literature focuses on the biochemical and clinical associations of elevated B12 [1-21]; however, there has not yet been an exploration of clinical awareness. This study represents the first, that we know of, survey of GPs and medical students on their knowledge of the topic. Our findings support the suggestion that elevated B12 is a commonly overlooked clinical finding [1,5,21,23]. The lack of formal teaching aligns with the absence of clinical guidelines. The qualitative comments from both groups provide context to this knowledge gap and highlight uncertainty regarding management, an area largely unaddressed by previous research.

Implications for research and practice

Given the absence of agreed pathways for investigating elevated B12, these findings suggest that clinicians may benefit from clearer educational resources. Our results are preliminary, yet show that further work is needed to determine which patients with hypercobalaminaemia actually warrant investigation and treatment. This is not least because elevated B12 can commonly be a benign finding [23], yet can also be associated with mortality and disease [1-16]. Furthermore, as elevated B12 is not an uncommon finding at the population level [23], this indicates that clinicians are likely to encounter hypercobalaminaemia with some regularity in routine practice. Further research could focus on validating investigation pathways in primary care, assessing which markers would be most effective to use to assess B12 deficiency, and evaluating the long-term outcomes of patients with persistent B12 elevation. Additionally, larger-scale research may find that educational interventions are valuable. A pragmatic clinical framework is ultimately required for GPs for this uncertain clinical finding.

## Conclusions

This pilot survey demonstrates a clear gap in clinician understanding of hypercobalaminaemia. While there was some awareness of disease associations among clinicians, we found inconsistency in investigative approaches and a lack of awareness of functional deficiency or laboratory artefacts. Medical students often did not have extensive knowledge regarding elevated B12, and their investigative approaches were reflective of this.

Given the absence of clear pathways for investigating and managing elevated B12, our pilot study gives justification for larger, multi-centred studies to develop and validate practical diagnostic pathways for primary care. Establishing clearer guidance and improving clinician awareness may help ensure that elevated B12 is interpreted appropriately and relevant underlying conditions are identified earlier.
